# Evidence for rapid ecological range expansion in a newly invasive plant

**DOI:** 10.1093/aobpla/plv038

**Published:** 2015-04-10

**Authors:** Silvia Matesanz, Tim Horgan-Kobelski, Sonia E. Sultan

**Affiliations:** 1 Área de Biodiversidad y Conservación. Departamento de Biología y Geología, Universidad Rey Juan Carlos, c/ Tulipán s/n, Móstoles 28933, Spain; 2 Biology Department, Wesleyan University, Middletown 06459, CT, USA

**Keywords:** Ecological distribution, invasive spread, light availability, phenotypic plasticity, soil moisture

## Abstract

Little is known about how an introduced species may expand its ecological range, i.e. the set of local environmental conditions in which it can successfully establish populations. Delimiting this range of conditions is a methodological challenge, because it is impossible to sample all potential field locations for any species in a given region. Developing approaches to track ecological range over time could substantially contribute to understanding invasion dynamics. In this study, we use a previously established sampling strategy to document apparent changes across a 15-year time interval in the ecological range of the Asian annual *Polygonum cespitosum* Blume in northeastern North America, where the species has recently become invasive. Using a structured sample drawn from a large set of field populations, we determined the range of light, soil moisture and soil nutrient conditions that the species currently occupies in this region and the proportional distribution of individuals in differing types of microsite, and compared them with field measurements that were similarly determined 15 years earlier. Although in 1994 the species was absent from both high-light and flooded habitats, in 2009 *P. cespitosum* occurred in open as well as shaded habitats, across a wide range of moisture conditions. In 2009 the species also occupied a greater proportion of high-light microsites within field sites than in 1994. These findings suggest an expanded ecological range that, intriguingly, is consistent with the recent evolution in North American *P. cespitosum* populations of adaptive plasticity in response to high light. Possible non-evolutionary explanations for the change in field distribution are also considered.

## Abstract (precis)

Many studies track the geographic spread of invasive plants, but little is known about how such species may expand the range of environments in which they can successfully establish populations-- the *ecological range.* This paper documents a significant expansion over a recent 15-year period in the range of light and moisture conditions inhabited by the Asian annual *Polygonum cespitosum* Blume in northeastern North America. The species' increased ecological breadth is coincident with its more aggressive spread in the region.

## Introduction

A non-indigenous species' transition from introduced to invasive is usually marked by the onset of aggressive range expansion (Theoharides and Dukes 2007; Valéry *et al*. 2008). Typically, studies of range expansion in introduced species model the geographic spread of these species at regional scales using historical presence/absence data, often in combination with climatic data (e.g. Elton 1958; Paterson 2000; Salo 2005; Preisser *et al*. 2008; Lyons and Scheibling 2009). However, within an introduced species' geographic range, ecological factors such as resource availability and community composition will determine which particular habitats the species is able to invade (Pearson and Dawson 2003). Despite the abundance of research documenting geographic range expansion in introduced species, little is known about how an introduced species may expand its ecological range, i.e. the set of local environmental conditions in which such a species can successfully establish populations (see Alexander and Edwards 2010; for a general discussion of species distribution in relation to ecological niche, see Guisan and Thuiller 2005; Hargreaves *et al*. 2014).

Ecological range expansion may be a particularly important aspect of the invasion process to document because it can influence future invasion dynamics in two key ways. First, expansion into a broader range of ecological conditions may enhance a species' rate of spread by increasing connectivity among suitable habitats and the probability of dispersal to suitable sites (With 2002; Hastings *et al*. 2005; Theoharides and Dukes 2007). Second, if newly colonized habitats have greater resource availability than ancestral habitats (which is typical of the disturbed, open conditions colonized by many invasive species, Theoharides and Dukes 2007), ecological range expansion may increase individual fitness, population growth rate and propagule pressure on adjacent habitats (Hastings *et al*. 2005; Lockwood *et al*. 2005).

A number of ecological and evolutionary processes can lead to changes in a species' ecological range (see Holt 2003). Expansion can occur after a species is introduced to a new region if its populations in that region evolve broader individual repertoires of adaptive plasticity to contrasting conditions (e.g. Lavergne and Molofsky 2007; reviewed in Richards *et al*. 2006; Matesanz *et al*. 2010). Indeed, selection in the disturbed, heterogeneous environments typically colonized by introduced species may favour the evolution of such increased plasticity, which may in turn allow an introduced species to spread into a wider range of habitats (Lee and Gelembiuk 2008; Sultan *et al*. 2013). Alternatively, ecological range expansion may occur if geographically separate populations become locally adapted to diverse habitats, if genotypes from distinct habitats in a species’ home range are brought in over time via multiple introductions (Dlugosch and Parker 2008), or due to non-evolutionary processes, such as changes in community composition that facilitate a introduced species' occupancy of previously unsuitable habitats (Hargreaves *et al*. 2014). For instance, populations of the Atlantic coast shore-grass *Spartina alterniflora* stabilize cobble beaches, which are then occupied by a diverse community of other plants (and their associated animals) that would otherwise be excluded (Bruno and Kennedy 2000).

Despite its importance for understanding biological invasion, determining the extent of a species' ecological range over time is problematic, since even a dataset based on many field populations may by chance lack infrequent types of sites that would enlarge the range. Indeed, the only *absolutely* definitive way to characterize a species' ecological range at a given time point is to sample every possible location within its geographic distribution and to measure all possible environmental parameters within each of them. A feasible alternative is to construct a set of natural populations, based on a broad sampling of field habitats, that collectively encompass the *full range of environmental conditions* (i.e. the highest and lowest resource levels) in which the species occurs and forms viable populations (Colwell and Futuyma 1971; see Sultan *et al*. 1998). This type of population sample, specifically designed to include the environmental extremes in which the species is found, will be more informative for delimiting ecological range than a random population sample (Sultan *et al*. 1998). Moreover, using a smaller number of field sites makes it possible to comprehensively measure environmental variability within each site and to assess the distribution of individuals among different microsites. Such microsite data are critical because they make it possible to distinguish environmental shifts in a species' realized distribution from changes that could possibly result simply from increased dispersal. A final element in this strategy is to focus on certain key environmental variables; such as light, soil moisture and nutrient availability. Using this approach at two or more time points, it is possible to statistically test for changes in ecological range for the specified variables. Despite the inevitable interpretive limitations of a finite field sample, such environmental comparisons can provide useful insights to this critical dimension of invasive spread.

Here we document a measurable change over a recent 15-year period in the ecological range of the Asian annual *Polygonum* (s.l.) *cespitosum* Blume (*Persicaria cespitosa*, Kim *et al*. 2008) in northeastern North America, where the species was introduced in the early 1900s (Paterson 2000). Its tiny achenes are readily dispersed (e.g. in mud and plant debris) and the species has spread rapidly across the continent (it has been reported throughout eastern North America), aided by transport on animal hooves, shoes and vehicle tyres. *Polygonum cespitosum* is of particular interest because it was rather recently introduced in New England (first listed in 1976; Mehrhoff *et al*. 2003) and subsequently has become invasive in this part of its non-native range. Throughout most of its residency in the region, the species has inhabited moderately shaded, moist sites (Sultan *et al*. 1998) similar to those typical of its native Asian range (Anjen *et al*. 2003). However, within just the past two decades, *P. cespitosum* populations have been reported in open sites in New England, often forming very dense monocultures (Mehrhoff *et al*. 2003). A series of glasshouse studies showed that, during this same time interval, local populations in the species' introduced range have rapidly evolved increased adaptive plasticity in response to high-light conditions (Sultan *et al*. 2013). This intriguing scenario suggests that the species might be undergoing an expansion of its ecological range to include open habitats, and that this change in ecological breadth could be contributing to its recent transition to invasiveness.

In this study, we measured the range of light, soil moisture and soil nutrient levels in which *P. cespitosum* currently occurs in northeastern North America (2009 field data), and compared these measurements with the species’ ranges of light, moisture and nutrient conditions that were similarly determined 15 years earlier (1994 field data; Sultan *et al*. 1998). Specifically, we asked the following questions: over the last 15 years, has *P. cespitosum* measurably expanded its ecological range in northeastern North America? If so, does the species occur in habitats from which it was absent in the past (e.g. sunny habitats), and has it shifted its occupancy of different types of microsite within sites? We discuss the potential implications of ecological range expansion on the species’ potential invasive spread, in terms of individual and population performance. Because detailed historical data on the environments inhabited by introduced species are rare, to our knowledge this is the first study that uses comparisons of environmental data across time to assess change in ecological range during an ongoing species invasion.

## Methods

### Population/site sample

In order to rigorously compare the recent (2009) ecological range of the species with its earlier (1994) ecological range, we precisely quantified the highest and lowest levels of insolation, soil moisture and macronutrient conditions in which viable populations of the species were found (see Colwell and Futuyma 1971). In 1994 and 2009, more than 50 field sites were examined in a common geographic area extending from southwestern Connecticut to northern and central Massachusetts (an area of ∼40 000 km^2^). This initial examination covered the complete range of habitat types in which annual plants occur in Northeastern North America, including a broad variety of naturally and artificially disturbed habitats such as forest paths and trails, road embankments and meadows. For each year, based on environmental measurements taken at 14–18 *P. cespitosum* sites chosen from this initial large sample (details below), five disjunct natural populations of the species, consisting of at least 100 individuals, were chosen to encompass the full range of conditions occupied. Hence, the final population sample in each year reflected the highest and lowest resource levels revealed in an initially large, random sample of field populations. Importantly, environmental conditions (daily precipitation for the months previous to data collection as well as annual precipitation) of the study years when the environmental measurements were performed were very similar (differences in spring–summer and annual precipitation between 1994 and 2009 were <4 %) **[see Supporting Information]** which allowed a meaningful comparison between years that was not affected by between-year climatic variation.

**Figure 1.**
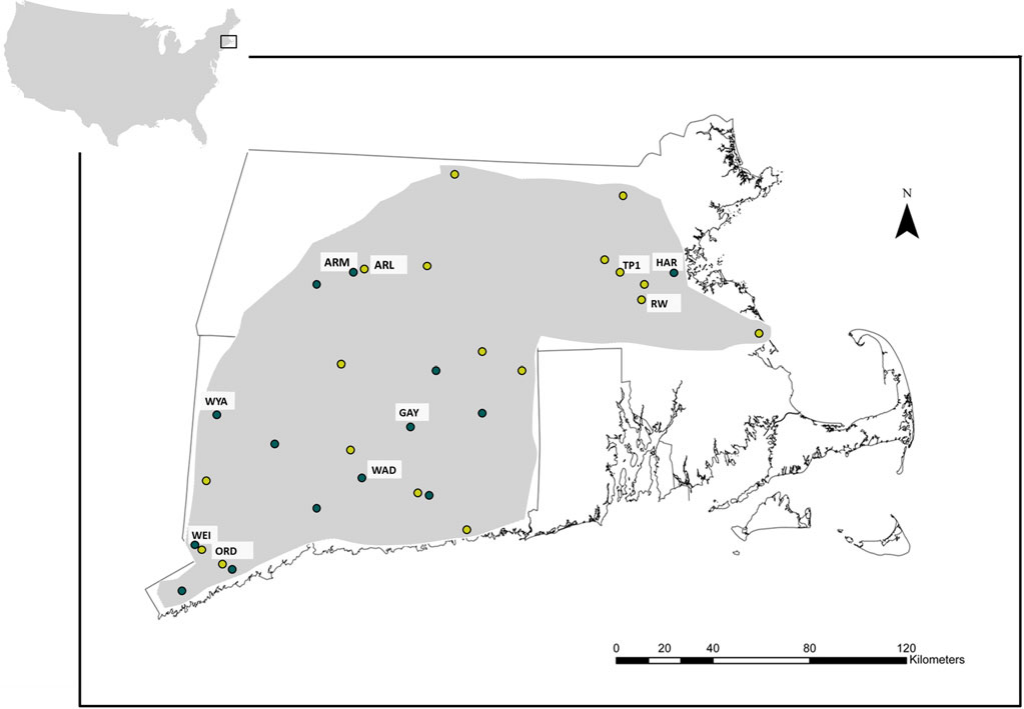
Location of field sites sampled in northeastern North America. The light grey area delimits the extent of the initial sampling area, which included more than 50 disjunct field sites encompassing all annual-plant habitats. The 18 yellow and 14 green dots represent the randomly chosen subset of sites measured in 1994 and 2009, respectively. Labelled dots represent the five sites used as the structured sample for each year, as explained in the Methods section. See **Supporting Information** for details on habitat types and environmental data for each population.

In 1994, light availability (% of full photosynthetically active radiation, PAR) and soil moisture (at 0–10 cm depth) were measured early in the growth season at 18 *P. cespitosum* population sites across the region (see details below and **Supporting Information**), and plant community composition. From this initial set of 18 populations, the five populations in sites most different from one another were selected for detailed investigation as representing the range of environmental conditions across which the species occurred. This subset included the sites with highest and lowest light availability (TP1 and ARL), highest and lowest soil moisture (TP1 and RW) and the sites with intermediate light and moisture conditions but contrasting mixtures of herbaceous and woody plant species (ORD and WEI; see Fig. 1 and **Supporting Information** for population locations and settings); details of measurements and population/site sample selection are available in Sultan *et al*. (1998). Importantly, these data were collected as part of a multi-species comparison, which included many sites in types of habitats from which *P. cespitosum* was absent, and therefore, any measured increase in ecological range between years was not likely due to incomplete sampling in 1994.

In 2009, a similar protocol was followed to sample 14 field populations across the region, and a subsample of five populations was identified that represented the species’ current ecological range (i.e. the sites with lowest and highest mean light availability, GAY and HAR), the sites with lowest and highest soil moisture (ARM and HAR) and two sites with intermediate values of both light availability and soil moisture and very different plant community composition (WAD and WYA) (details in Fig. 1 and **Supporting Information**). See **Supporting Information** for environmental data for all fourteen 2009 populations and Sultan *et al*. (1998) for data on the 1994 populations.

### Environmental measurements

To determine the species' environmental distribution among as well as within sites, we collected environmental data at the five representative population sites in each year. In order to further ensure comparability of the environmental measurements in both years, the same field protocols were followed (see below). For both years, field measurements were made both (i) during seedling establishment and juvenile growth (23 June–6 July 1994 and 28 June–15 July 2009; early sampling date) and (ii) during peak of achene (seed) production by mature plants (25 August–3 September 1994 and 31 August–15 September 2009; late sampling date). In both 1994 and 2009 the early sampling date was well after tree leaf expansion, and the late sampling date was before the onset of autumn leaf fall, so measurements were taken at comparable points in the growth season across years.

#### Soil moisture

Soil moisture (percentage of field capacity) was determined gravimetrically at both early and late sampling dates for each population site, from samples taken at 0–10 cm depth and 20–30 cm depth. A soil core sampler was used to collect individual samples from 8 to 10 evenly spaced microsites per soil depth per population roughly evenly spaced across the spatial extent of the site area. To ensure comparable sampling conditions, all samples were collected at least 3 days after the most recent rainfall. Soil moisture content was calculated as the percentage of soil mass lost after oven-drying at 70 °C to constant weight. Soil moisture content was then converted to a percentage of field capacity (a proxy for soil water potential) which was determined separately for each site soil and sample depth by weighing, drying at 65 °C for 48 h, and reweighing soil that had been fully saturated with water and allowed to drain overnight (Klute 1986).

#### Light availability

Instantaneous light availability was measured on sunny days between 1030 and 1600 hours using an Accupar LP-80 sunfleck ceptometer (Decagon Devices, WA, USA). Each data point consisted of the mean of 10 adjacent PAR sensors spaced 1 cm apart along a 0.8 m transect, giving eight data points per transect. At each population site, on each sampling date, a total of 15–16 transects covering the spatial extent of the population were established at both *Polygonum* canopy height (∼20–30 cm aboveground level) and one-half of canopy height (10–15 cm), for a total of 240–256 data points per population site per date (8 data points per sample × 15–16 samples per population × 2 heights). Measurements were converted to a percentage of total available PAR in full sun (PAR intensity at a separate quantum sensor placed in the nearest fully insolated location and wired to the ceptometer). To characterize the distribution of high- and low-light microsites, the proportion of measurements with >80 % available PAR and <10 % available PAR was calculated for each sample of eight measurements from each transect for each site and date.

In 2009, the species’ range of light conditions was also quantified using hemispherical photography, a method of directly quantifying canopy closure to infer light availability based on the sun's trajectory. At each population site, at both early and late sampling dates, 15 photographs spaced >1 m apart were taken at *Polygonum* canopy height with a Nikon Coolpix 900 camera and Nikon FCE8 180° hemispherical lens (Nikon, Tokyo, Japan). A hand-held level and magnetic compass were used to orient the camera. Photographs were analysed with HemiView version 2.1 (Delta-T Devices, Ltd, Cambridge, UK), with light/dark thresholds selected based on the photograph's lighting and individually checked for each image to ensure consistent analysis. Global site factor (GSF), an indicator of photosynthetic photon flux density (PPFD) received during a day compared with PPFD at a completely unobstructed site, was calculated as (0.9 × direct site factor + 0.1 × indirect site factor) (Valladares *et al*. 1997). Mean and median sunfleck durations (interval of direct insolation) were calculated for each photograph. Calculations of the Sun's trajectory were made for 1 July (early sampling date photographs) and 25 August (late sampling date photographs). Comparisons of the percentage of total available PAR calculated from the *in situ* measurements to the mean GSF for each site calculated with hemispherical photography rendered very similar results.

#### Soil properties

Soil pH, nutrient content and structural properties were determined from six soil samples per population site, depth (0–10 cm or 20–30 cm) and sampling date, sampled from across each population site as for soil moisture (see above; *N* = 24 per site). Samples from the same population, depth and sampling date were combined for analysis (four combined samples per site). Soil tests were performed at the University of Massachusetts Amherst Soil and Plant Tissue Testing Lab (http://www.umass.edu/plsoils/soiltest/) using the Morgan extraction procedure to generate site, depth and seasonal means for: pH, cation exchange capacity (CEC, meq/100 g) and concentrations of phosphorus (ppm), potassium (ppm), calcium (ppm), magnesium (ppm) and nitrate ($\text{N}{{\text{O}}_{3}}^{-},$ ppm). Soil organic content was determined as the percentage of mass lost upon ignition.

#### Plant size and demography

In 2009, for each population we measured *Polygonum* individual plant height, leaf number and reproductive output, as well as population density, cover and phenology (proportion of flowering individuals) at both early and late sampling dates. At each sampling date, 10 circular plots (0.5 m diameter) were haphazardly selected across the population. In each plot, all leaves and inflorescences were counted from a single, randomly selected *Polygonum* plant. Also, all *Polygonum* flowering and non-flowering individuals were counted, and *Polygonum* cover was estimated by two independent observers. At the late sampling date, all inflorescences from a single plant in each plot were collected, air-dried and weighed to estimate total individual reproductive output. Reproductive output per square metre was estimated as individual reproductive output × population density (based on the late sampling date measurements).

### Data analysis

Mixed ANOVA with type-III sums of squares was used to test for differences between sampling years in soil moisture (% of field capacity), light availability (% available PAR) and the proportion of low- and high-light microsites. The model tested for the effects of year (2009 vs. 1994), population site (nested within year) and sampling date (early vs. late) for all variables, and also for the effect of height (canopy vs. mid-canopy) for light measurements, depth (0–10 cm vs. 20–30 cm) for soil moisture measurements and ceptometer transect (nested within year, population, sampling date and height) for percentage available PAR. Measurement height (or depth) and sampling date were included as terms in the analysis because they were of interest as distinct samples of different aspects of the population's environment, though separate analyses conducted with data from the different depths or sampling dates as unique datasets rendered similar results. Transect was treated as a random factor. All other factors were treated as fixed. Initially, all interactions were tested, but because the interactions were not of primary interest, interactions for which *P*> 0.2 in the initial analysis were dropped from subsequent analyses. Because depth was NS for soil moisture, the two depths were pooled in correlation analyses (see below). Because soil samples for nutrient analysis were pooled from each site, depth and sampling date, we did not statistically test for differences among populations in soil properties. Mean nutrient content values were calculated for each population in each year, and these population-mean values for 1994 and 2009 were categorized according to current guidelines for soils in this region (University of Connecticut Soil Testing Lab, http://soiltest.uconn.edu/factsheet.php, University of Massachusetts Soil and Plant Tissue Testing Lab, http://soiltest.umass.edu/fact-sheets/soil-test-interpretation-recommendations).

To test for ecological range expansion, linear contrasts were used comparing the most extreme site in 1994 to the most extreme site in 2009 for each factor (e.g. the contrast tested for a significant difference between the driest site in 1994 and the driest site in 2009, etc.). In cases where the contrast showed a significant change in the most extreme site for a given environmental factor, the second most extreme site from the year with the broader range was compared with the most extreme site from the year with the narrower range to verify that the measured change in ecological range was not due to sampling an extremely unusual site by chance. Linear correlation analyses were used to assess the correlations of individual and population performance (leaf number, reproductive output, density, cover, proportion of individuals flowering, reproductive output per square meter) with environmental variables (soil moisture and GSF) in 2009. All analyses were performed in JMP v 7 (SAS Institute, Cary, NC, USA).

## Results

### Soil moisture

**Figure 2.**
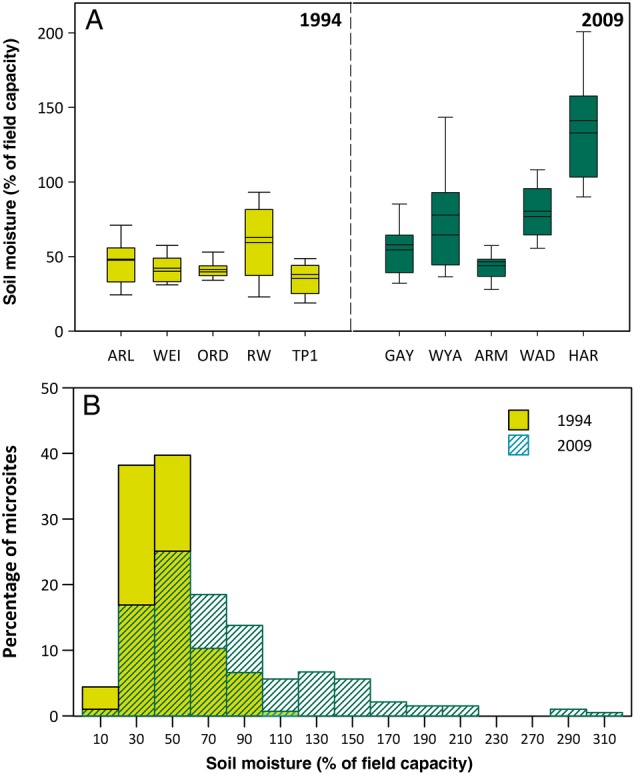
Expansion in soil moisture conditions occupied by *P. cespitosum* in its introduced range. (A) Box plots (10th, 25th, 50th, 75th and 90th percentile) and means (dark line) for soil moisture (% of field capacity) at five populations representing *P. cespitosum*'s ecological range in northeastern North America in 1994 (left) and 2009 (right). (B) The percentage of microsites by soil moisture availability in 1994 and 2009. Data from both depths and sampling dates were pooled.

In 2009, *P. cespitosum*'s ecological range included sites with higher average soil moisture than those it inhabited in 1994, as well as wetter microsites (Table 1; Fig. 2). In 1994, mean soil moisture at *P. cespitosum* populations (% of field capacity for each site's soil) ranged from 35 to 59 %, while in 2009 mean soil moisture ranged from 47 % to over 140 % (Fig. 2A). Both the wettest and second wettest 2009 sites had significantly higher mean soil moisture than the wettest site from 1994 (linear contrasts *P* ≤ 0.001 and *P* ≤ 0.006 respectively; Fig. 2A). Similarly, *P. cespitosum* inhabited a wider range of soil moisture microsites in 2009 than in 1994. Whereas in 1994, 95 % of all microsites sampled in *Polygonum* populations had soil moisture between 19 and 93 % of field capacity, in 2009 this 95 % microsite range extended from 23 to 206 % of field capacity (Fig. 2B). Surprisingly, 25 % of all microsites sampled in 2009 had higher soil moisture than any microsite sampled in 1994. The difference in mean soil moisture between the driest site in 1994 (35 % of field capacity) and in 2009 (47 % of field capacity) was not significant (*P* = 0.136).

**Table 1.** Top: effects of year, site, sampling date and depth on soil moisture availability in introduced-range populations of *P. cespitosum*. Bottom: effects of year, site, sampling date and measurement height on light availability in introduced populations: (A) mean light availability, (B) percentage of low-light microsites and (C) percentage of high-light microsites. Degrees of freedom, *P-*values and MS are shown from mixed-model ANOVA (for details, see Methods). Bold values are significant at *P* < 0.05.

Soil moisture availability											
	Df	MS	*P*								
Year	1	1010	**<0**.**001**								
Site (Year)	8	277.6	**<0**.**001**								
Sampling date	1	7.084	**0**.**0403**								
Depth	1	5.496	0.461								
Date × depth	1	20.61	0.154								
Year × date × depth	1	21.88	0.142								
Error	317	10.09									
**Light availability**											
**Mean light availability**	**Df**	**MS**	***P***	**% of low-light microsites**	**Df**	**MS**	***P***	**% of high-light microsites**	**Df**	**MS**	***P***
Year	1	209.1	**<0**.**001**	Year	1	33.46	**<0**.**001**	Year	1	17.16	**<0**.**001**
Site [Year]	8	54.41	**<0**.**001**	Site [Year]	8	18.76	**<0**.**001**	Site [Year]	8	4.777	**<0**.**001**
Sampling date	1	12.05	0.104	Sampling date	1	0.3891	0.588	Sampling date	1	2.417	**0**.**036**
Height	1	95.99	**<0**.**001**	Height	1	18.91	**<0**.**001**	Height	1	18.10	**<0**.**001**
Year × date	1	20.77	**0**.**033**	Year × date	1	9.251	**0**.**008**	Year × date	1	1.228	0.134
Site × date	8	17.36	**<0**.**001**	Site × date	8	8.522	**<0**.**001**	Year × height	1	3.807	**0**.**009**
Transect	553	4.54	**<0**.**001**	Year × date × height	1	2.508	0.169	Site × date	8	1.670	**0**.**002**
Error	4170	0.1309		Error	571	1.321		Site × height	8	1.073	**0**.**049**
								Date × Height	1	1.887	0.064
								Error	562	0.5463

### Light availability

**Figure 3.**
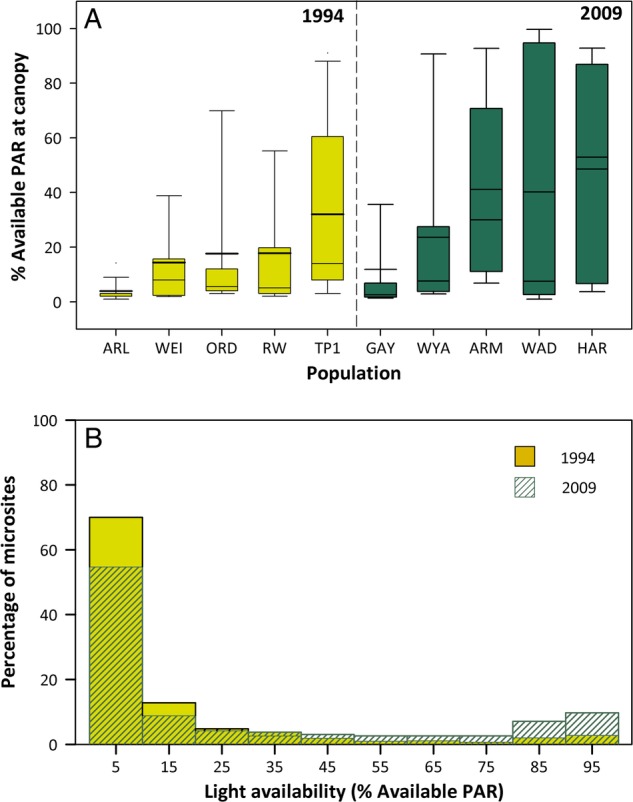
Expansion in light availability conditions occupied by *P. cespitosum* in its introduced range. (A) Box plots (10th, 25th, 50th, 75th and 90th percentile) and means (dark line) for light availability at canopy height (% available PAR) at five populations representing *P. cespitosum*'s ecological range in northeastern North America in 1994 and 2009. (B) The percentage of microsites by light availability in 1994 and 2009. Data from both sampling dates were pooled.

In 1994, mean light availability at *P. cespitosum* populations ranged from 5 to 32 % at canopy height, and from 2 to 17 % at mid-canopy height (Fig. 3A). In 2009, mean light availability ranged from 12 to 49 % at canopy height and from 7 to 34 % at mid-canopy. Both the site with highest mean light availability (‘sunniest population’) and second sunniest 2009 populations had significantly higher mean light availability than the sunniest site in 1994 (*P* ≤ 0.001 and *P* ≤ 0.007, respectively). In contrast, there was no significant difference in mean light levels between the site with lowest mean light availability (‘shadiest population’) in 1994 and the shadiest population in 2009 (*P* ≤ 0.143). In both 1994 and 2009, plants occurred in very low-light microsites (<10 % available PAR at canopy height) as well as high-light microsites (>80 % available PAR at canopy height) (Fig. 3B), but in 2009 a lower proportion of plants occurred in low-light microsites (55 vs. 70 %), and a higher proportion of plants were found in high-light microsites (17 vs. 5 %) (Fig. 3B; effect of year in Table 1 bottom).

**Figure 4.**
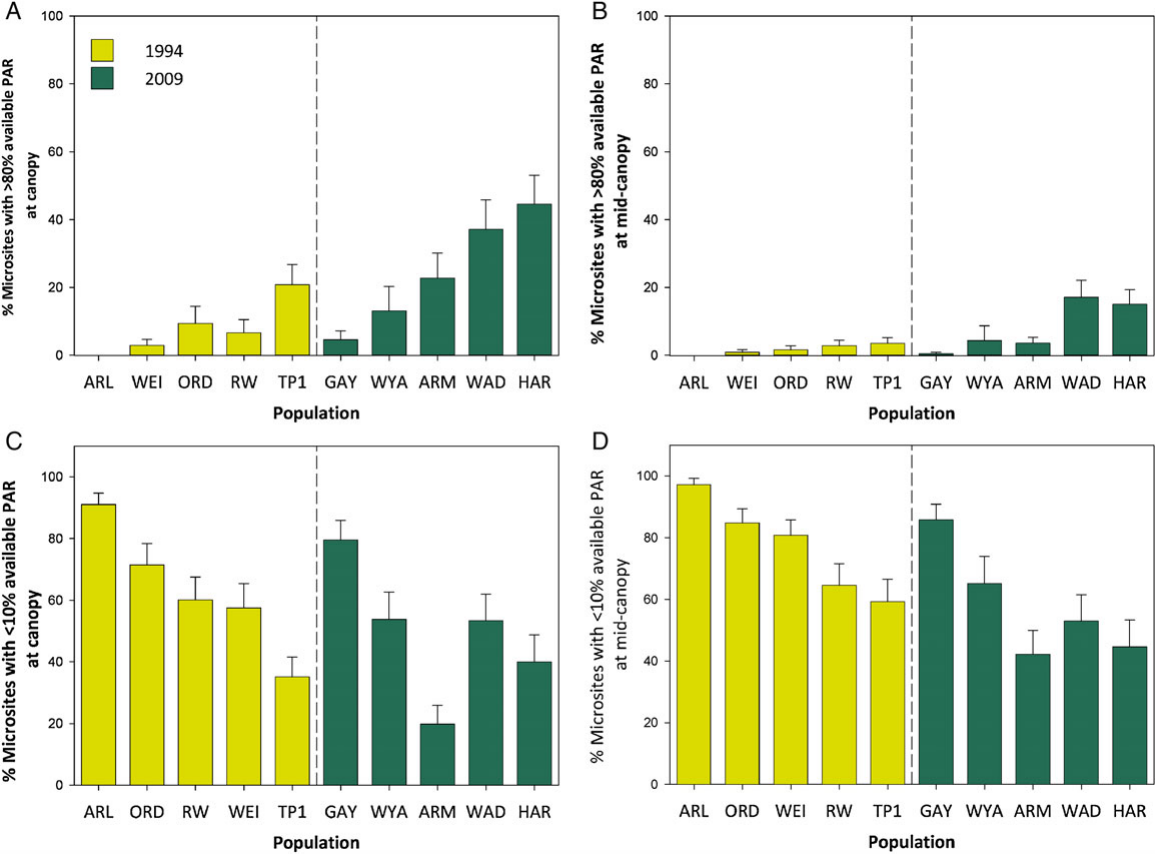
Changes in the frequency of high- and low-light microsites in the introduced range of *P. cespitosum*. Shown are means + 1SE at five populations representing *P. cespitosum*'s ecological range in northeastern North America in 1994 and 2009 for (A) the percentage of high-light microsites at canopy height, (B) the percentage of high-light microsites at mid-canopy height, (C) the percentage of low-light microsites at canopy height, and (D) the percentage of low-light microsites at mid-canopy height. Data from both sampling dates are pooled (for details, see Methods).

Both the sunniest and second sunniest sites in 2009 had a significantly higher proportion of high-light microsites than the sunniest site from 1994 (*P* ≤ 0.001 and *P* ≤ 0.001); in 1994, the sunniest site had 21 and 4 % of microsites, >80 % PAR, at canopy and mid-canopy height, respectively, compared with 45 and 15 % of microsites, respectively, at the sunniest site in 2009 (Fig. 4A and B). The shadiest sites from 1994 and 2009 did not differ significantly in the proportion of high-light microsites (*P* ≤ 0.552); at the shadiest population in 1994, no microsites at either canopy or mid-canopy level received >80 % of available PAR, while in 2009, at the shadiest site 5 % of microsites at canopy height and 0.4 % of microsites at mid-canopy height received >80 % PAR (Fig. 4A and B). Neither the sunniest sites from both years nor the shadiest sites from both years differed significantly in the proportion of low-light microsites (*P* ≤ 0.447 and *P* ≤ 0.081, respectively).

### Soil pH and nutrients

In both years, *P. cespitosum* populations were found in sandy loam soils that included a very broad range of soil nutrient levels. The range of soil pH and nutrient values was generally similar in 1994 and 2009 (5.1–7.4 and 5.1–6.7 in 1994 and 2009, respectively). In both years, population-mean CEC values fell within the moderate-optimal range and were typical for soils found in the study region (1.8–18.0 and 3.8–19.0 meq/100 g in 1994 and 2009, respectively). Ranges of values for individual soil nutrients were also broadly similar between 1994 and 2009: nitrate concentration was low to moderate in both 1994 and 2009 (5.4–23.6 and 10.0–15.0 ppm); Potassium levels ranged from low to high in 1994 (56–192 ppm) but were low in 2009 (55–72 ppm); calcium levels ranged from very low to optimum in both years (320–1895 and 246–1336 ppm in 1994 and 2009, respectively). Magnesium content ranged from very low to above optimal in both years (27–303 and 26–180 ppm in 1994 and 2009, respectively), and phosphorous contents ranged from very low to either optimal or excessive in both years as well.

### Community composition

*Polygonum cespitosum* populations were found in a broad range of plant community types in both study years. In 1994, plant communities included predominantly annual species, mixed forest understory and diverse mixtures of perennials, broadleaf and coniferous woody plants. Similarly, in 2009, plant communities included evergreen and deciduous forest understories as well as mixed annual, perennial and woody species.

### Population performance

At the late sampling date in 2009, population-mean soil moisture was positively correlated with mean leaf number (*r* = 0.848, *P* = 0.002), mean inflorescence number (*r* = 0.880, *P* ≤ 0.001) and mean reproductive output (*r* = 0.864, *P* ≤ 0.001); and negatively correlated with mean population density (*r* = 0.576, *P* = 0.081). These correlations were disproportionately influenced by one population with very high soil moisture (see Fig. 2A), and were no longer significant (*P* > 0.2) when this population was removed from the analysis.

Light availability (quantified as GSF) was positively correlated with several indicators of individual and population performance. At the early sampling date, population-mean GSF was positively correlated with the proportion of individuals flowering (*r* = 0.800, *P* ≤ 0.001), mean plant height (*r* = 0.866, *P* ≤ 0.001) and mean leaf number (*r* = 0.658, *P* = 0.014). At the late sampling date, population-mean GSF was positively correlated with mean plant height (*r* = 0.937, *P* ≤ 0.001), mean leaf number (*r* = 0.769, *P* = 0.009), mean inflorescence number (*r* = 0.667, *P* = 0.035) and mean reproductive output (*r* = 0.729, *P* = 0.017), as well as mean *Polygonum* cover (*r* = 0.578, *P* = 0.080) and mean reproductive output per unit area (*r* = 0.758, *P* = 0.011). See **Supporting Information** for plant size, demography, phenology and reproductive output data of the 2009 populations.

## Discussion

A study of comparable multi-population field datasets indicates that in the 15 years between 1994 and 2009, the introduced Asian annual *P. cespitosum* measurably expanded its ecological range in northeastern North America. In 1994, the species was excluded from high-light as well as flooded habitats and habitat patches (microsites within sites) in this region (Sultan *et al*. 1998). As of 2009, the distribution of *P. cespitosum* in the same region included sites with increased mean light availability, a higher proportion of high-light microsites, sites with higher average soil moisture and wetter microsites than were occupied by the species in 1994.

The newly colonized high-light habitats span the range of moisture environments inhabited by *P. cespitosum.* Although the driest sites in both years had similar soil moisture, the driest site in 2009 had higher light availability than the driest site in 1994. Because of the increased transpirational demands of high-light habitats, the combination of high light and dry soil represents a novel stress level for the species compared with its 1994 distribution. The change in *P. cespitosum*'s distribution documented in this study signifies a true expansion since the species now colonizes the full breadth of 1994 habitats as well as the new, sunnier and wetter habitats. Although this initial dataset is not definitive, these findings suggest that ecological range expansion is accompanying the species' increasingly invasive spread in the region (Mehrhoff *et al*. 2003).

Comparative data on field distribution cannot be absolutely conclusive, since one cannot exhaustively sample every possible site across a large range. However, the difference described above is unlikely to be an artefact for several reasons. First, the 1994 data on *P. cespitosum*'s ecological range were collected as part of a multi-species comparison that included annual congeners that did occupy high-light and flooded habitats and habitat patches. Although many such sites in the region were examined in 1994, *P. cespitosum* was not found in *any* open, moist site or habitat patch within a site, which strongly supports the conclusion that *P. cespitosum* did not occur in these conditions in northeastern North America at that time (Sultan *et al*. 1998). Second, our sampling strategy allowed us to assess the distribution of *P. cespitosum* plants with respect to environmentally distinct microsites within each of the field populations, a key indicator of change in the species' realized ecological niche. Compared with the 1994 dataset, in 2009 a significantly lower proportion of plants occurred in low-light microsites, and a (more than three-fold) higher proportion were found in high-light microsites. Although it could perhaps be argued that the species' new occupancy of open, wet sites might reflect newly successful dispersal into those sites rather than a change in ecological tolerance *per se*, a changed pattern of microsite occupancy *within* field sites could not result from greater dispersal (see Hargreaves *et al*. 2014 on the difficulty in many distribution studies of distinguishing dispersal from environmental factors).

Finally, annual precipitation in this area was virtually identical in 1994 and 2009, so the difference in soil moisture documented in the 2009 *P. cespitosum* sites and microsites does not reflect between-year climatic variation. As with any multi-year field study, however, the possibility remains that some unknown environmental difference between measurement years contributed to the change here documented in the extent of resource levels occupied by the species. For instance, if a competitor, pathogen or herbivore, was present in high-light and wet sites in 1994 but absent for some reason in 2009, the species might be newly able to occupy those sites. It seems unlikely however that any such ecological interaction would be consistently present versus absent in all of the numerous sites sampled each year. A species' ecological distribution can also shift or expand due to changes within habitats in the species' geographical range, for instance in community composition, soil biota or disturbance regime. The change between 1994 and 2009 in *P. cespitosum*'s ecological distribution within northeastern North America does not appear to be related to such changes within that range, since we found no changes in the presence or abundance of interacting plant or animal species. Instead, the species is now colonizing open habitat types that were already present in this part of its introduced range (and indeed already inhabited by congeners such as *P. persicaria*; Sultan *et al*. 1998). This suggests that ecological range expansion may have resulted from a change in the species' performance in high-light environments, rather than from environmental changes at *P. cespitosum* populations. However, it is possible that changes in microbial community that we did not measure could have also contributed to the changed ecological range of the species.

Along with these possible habitat factors influencing ecological distribution, broad environmental tolerance that results in an invasive species colonizing a variety of habitats may result from high individual plasticity (Sultan 2004). Indeed, many successful invasive species show greater plasticity for functional traits in the introduced compared with the native range (Richards *et al*. 2006). An intriguing possibility is that ecological range expansion in *P. cespitosum* between 1994 and 2009 may have been facilitated by changed patterns of adaptive plasticity. Such plasticity changes were found to have occurred between 1994 and 2005 in three replicate North American populations of the species (Sultan *et al*. 2013). Compared with earlier-collected (1994) individuals, recently evolved (2005) *P. cespitosum* plants showed greater functional adaptation and significantly higher fitness in a glasshouse treatment simulating the high-light, high-moisture environments *P. cespitosum* has recently begun to colonize, but no reduction in performance in controlled shade conditions (Sultan *et al*. 2013). Although a retrospective field study such as this cannot definitively test any causal explanation, it is certainly suggestive that these rapidly evolved changes in individual plasticity patterns are broadly consistent with the expansion in *P. cespitosum*'s ecological distribution. The coincidence of rapid evolution of adaptations to high-light environments, and the species' spread into high-light habitats in northeastern North America during the same very recent time period, suggests that the evolution of increased plasticity in introduced-range populations of *P. cespitosum* may have contributed to the ecological range expansion of the species in this region.

Whatever the precise causes, the species' expanded ecological range is likely to affect the future dynamics of its invasive spread. Clearly its geographic spread will be increased if a greater proportion of the introduced range offers suitable habitat. Additionally, expansion into new sites will result in increased connectivity among suitable habitats, which is likely to enhance dispersal to suitable sites and the rate of spatial spread (With 2002; Hastings *et al*. 2005; Theoharides and Dukes 2007). This may be particularly important for *P. cespitosum*'s spread in northeastern North America, where the species' historic low-light forest habitats are interspersed with the open meadows, agricultural fields and developed areas it is now able to colonize. Increased habitat connectivity will also enhance gene flow among populations, which may affect processes like local adaptation and geographic expansion at the leading edge of the invasion (Theoharides and Dukes 2007). *Polygonum cespitosum* populations in the newly colonized high-light sites showed higher reproductive output at both individual and population levels compared with populations in sites with lower levels of available light. This enhanced performance may lead to further expansion into new sites and possibly new habitats as well, as a result of increased propagule pressure on adjacent locations (With 2002; Hastings *et al*. 2005; Lockwood *et al*. 2005).

Niche-based models of species' distributions in future environments (including habitat-distribution and climate-envelope models) usually assume constant habitat requirements through time (e.g. Peterson and Vieglais 2001). Our results provide initial empirical support for the possibility that a species’ ecological range may actually expand, perhaps rapidly (see also Pearson and Dawson 2003; Alexander and Edwards 2010). A research focus on ecological range can add a critically important dimension to the study of biological invasion as well as species' distributions more broadly (see meta-analysis in Hargreaves *et al*. 2014). We hope that this initial study will encourage invasion biologists to develop robust, consensus approaches to the challenging but central issue of ecological range expansion.

## Sources of Funding

This work was funded by a Marie Curie IOF Postdoctoral Fellowship (European Commission FP7) and a US National Science Foundation Research Grant (DEB-9496050). T.H.-K. was supported by Wesleyan University, and undergraduate assistants (1994) were funded by the Howard Hughes Medical Institute.

## Contributions by the Authors

Field data were collected by T.H.-K. and S.M. (2009), and by S.E.S. (with assistance from S. D. Hann and B. J. Brosi) (1994). All authors contributed substantially to manuscript preparation.

## Conflict of Interest Statement

None declared.

## Supporting Information

The following additional information is available in the online version of this article –

**Table S1.** Study population locations, habitat description and soil moisture and light availability data (10–90 % percentiles).

**Figure S1.** Long-term annual rainfall in the study region (statewide averages for Connecticut, top and Massachusetts, bottom).

**Figure S2.** Daily rainfall in the study region in 1994 (top) and 2009 (bottom), for the months immediately before data collection.

**Figure S3.** *Polygonum cespitosum*'s ecological range in northeastern North America in 2009.

**Figure S4.** Plant height, leaf number, % individuals flowering, density, relative cover and reproductive output in populations in northeastern North America in 2009, both in the early and late sampling dates.

Additional Information
