# Supplementary material for: Evidence for rapid ecological range expansion in a newly invasive plant
Source: AoB Plants. 2015 Apr 10;7:plv038. doi: 10.1093/aobpla/plv038 (PMC4511186; doi:10.1093/aobpla/plv038)
Supplement: Additional Information [file supp_7_plv038_index.html]

Evidence for rapid ecological range expansion in a newly invasive plant — Evidence for rapid ecological range expansion in a newly invasive plant — Additional Information 

# Evidence for rapid ecological range expansion in a newly invasive plant

## Additional Information

Additional Information

**Files in this Data Supplement:**

- Additional Information - Docx file
