# Supplementary material for: Evidence for rapid ecological range expansion in a newly invasive plant
Source: AoB Plants. 2015 Apr 10;7:plv038. doi: 10.1093/aobpla/plv038 (PMC4511186; doi:10.1093/aobpla/plv038)

**Supporting Information 1.** Study population locations, habitat description and soil moisture and light availability data (10-90% percentiles). *Polygonum cespitosum*’s distribution in 1994 and 2009 is represented by the five most different population sites chosen from a larger set of populations in each year (details in Methods).

|  | **Population** |  | **Habitat Description** | **Soil moisture range (%)** | **Light availability range (%)** | **Location** |
| --- | --- | --- | --- | --- | --- | --- |
|  |  |  |  |  |  |  |
| **1994 Populations** | ARL |  | Shaded, moist bank | 25-67% | 1-9% | Leeds, MA |
|  |  |  |  |  |  |  |
|  | ORD |  | Shaded meadow | 34-53% | 3-66% | Weston, CT |
|  |  |  |  |  |  |  |
|  | RW |  | Shaded, moist trail | 24-93% | 2-55% | Medfield, MA |
|  |  |  |  |  |  |  |
|  | WEI |  | Roadside embankment | 32-57% | 2-38% | Wilton, CT |
|  |  |  |  |  |  |  |
|  | TP1 |  | Shaded horse paddock | 19-48% | 3-88% | Dover, MA |
|  |  |  |  |  |  |  |
| **2009 Populations** | GAY |  | Shaded trail | 32-85% | 1-35% | Hebron, CT |
|  |  |  |  |  |  |  |
|  | WYA |  | Shaded path | 37-141% | 3-90% | Kent, CT |
|  |  |  |  |  |  |  |
|  | WAD |  | Horse path and clearing | 56-108% | 7-93% | Middletown, CT |
|  |  |  |  |  |  |  |
|  | ARM |  | Roadside embankment | 28-57% | 1-99% | Leeds, MA |
|  |  |  |  |  |  |  |
|  | HAR |  | Moist, lowland clearing | 90-200% | 4-93% | Jamaica Plain, MA |

**Supporting Information 2.** Long-term annual rainfall in the study region (statewide averages for Connecticut, top and Massachusetts, bottom). Highlighted in red are the years when *Polygonum cespitosum* ecological range was assesed. Data obtained from the National Climatic Data Center of the National Oceanic and Atmospheric Administration (NOOA; [www.ncdc.noaa.gov](http://www.ncdc.noaa.gov)).





**Supporting Information 3.** Daily rainfall in the study region in 1994 (top) and 2009 (bottom), for the months immediately before data collection. Data obtained from the National Climatic Data Center of the National Oceanic and Atmospheric Administration (NOOA; [www.ncdc.noaa.gov](http://www.ncdc.noaa.gov)), for the station Hartford Brainard Field (CT USA), which is located roughly at the center of the study area.

**
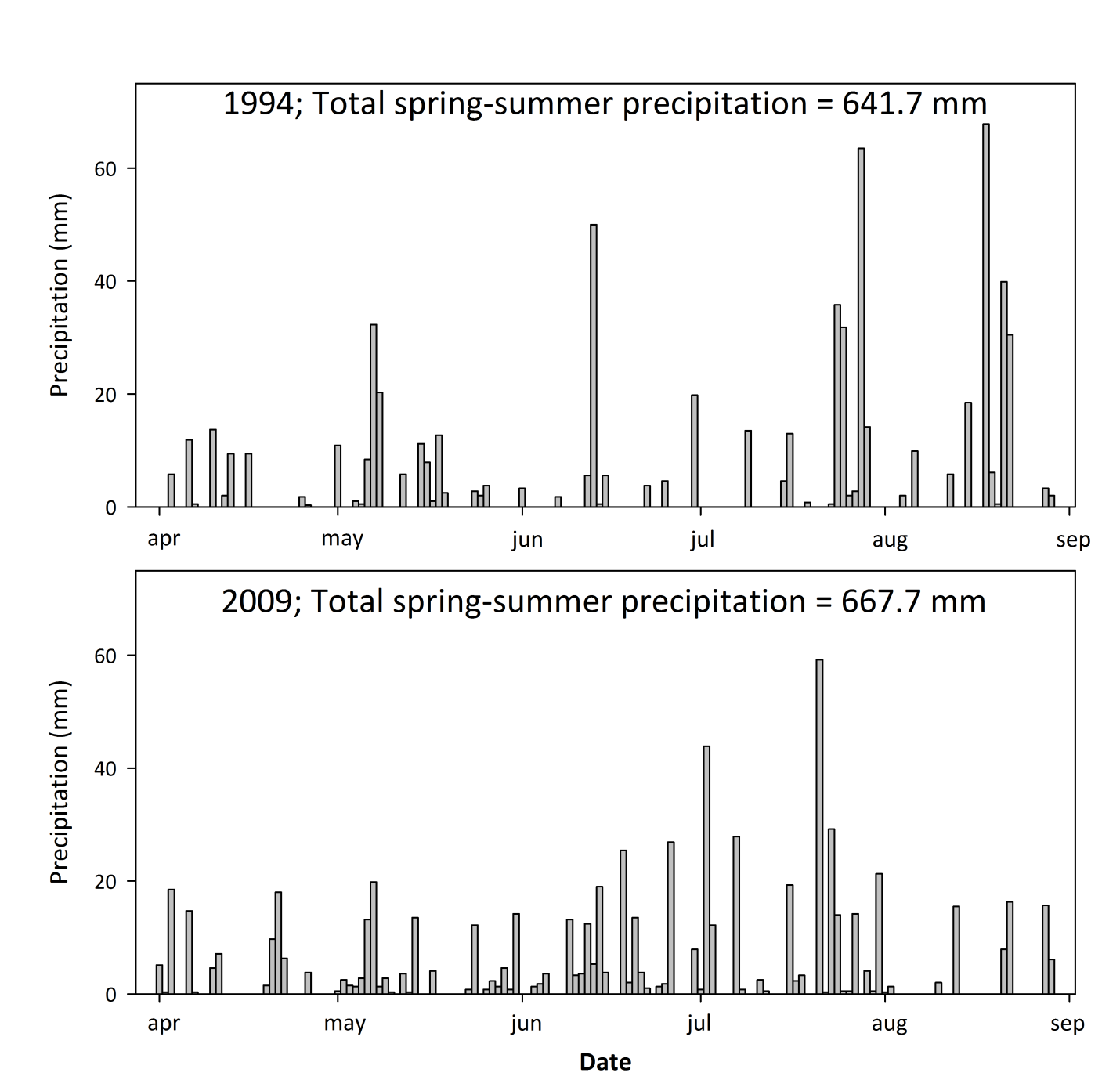
**

**Supporting Information 4.** *Polygonum cespitosum*’s ecological range in northeastern North America in 2009. Shown are (a) population means and data ranges for soil moisture, (b) population means + 1SD for mean sunfleck duration, (c) Global Site Factor (mean and data ranges), and (d) population median and quartiles (25%-75%) for sunfleck duration, for the full set of 14 populations measured in 2009.





**Supporting Information 5.** Plant height, leaf number, % individuals flowering, density, relative cover and reproductive output in populations in northeastern North America in 2009, both in the early and late sampling dates.


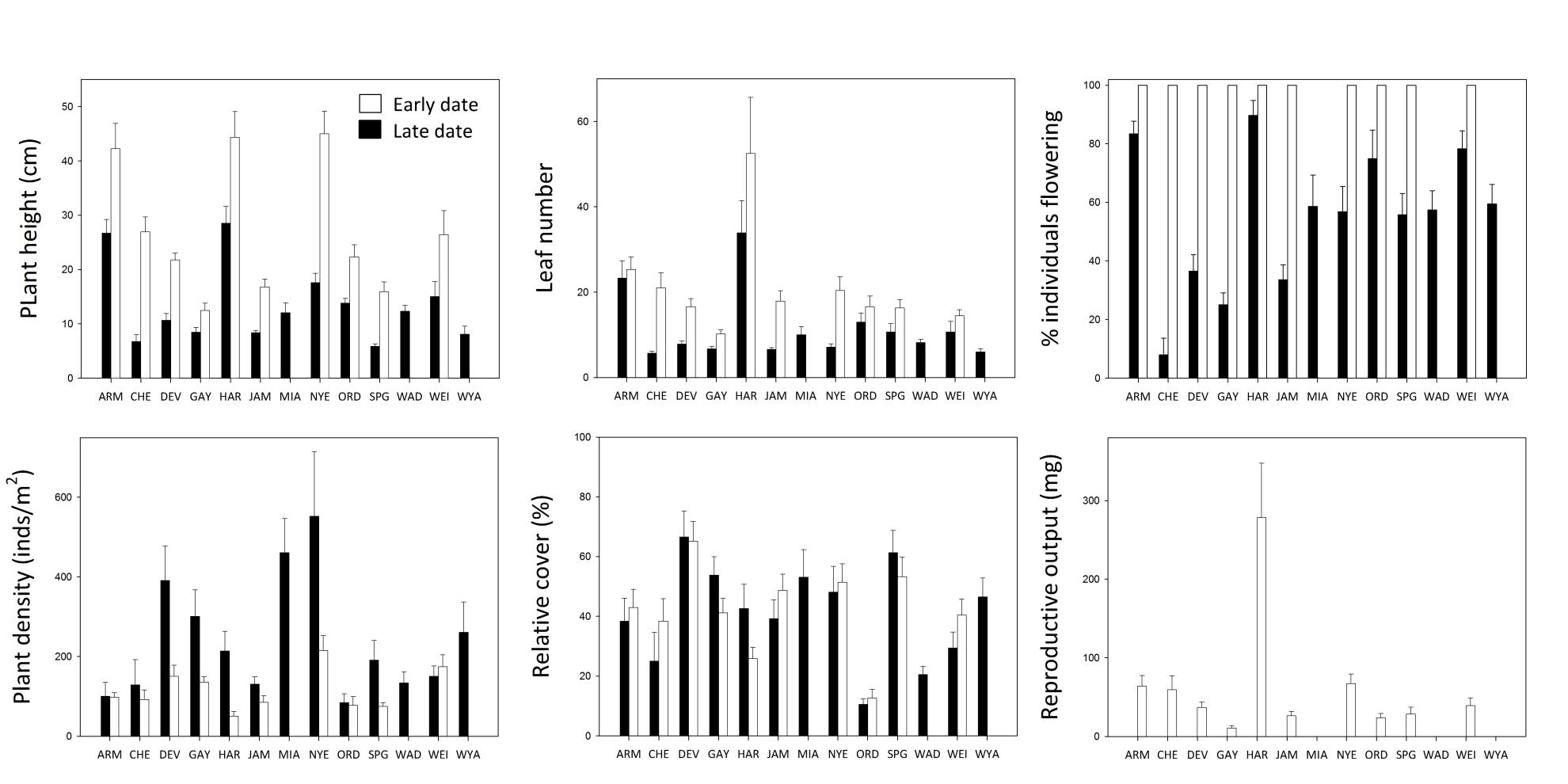

Supplement: Additional Information [file supp_plv038_plv038supp.docx]
